# Dendritic Cells: A Bridge between Tolerance Induction and Cancer Development in Transplantation Setting

**DOI:** 10.3390/biomedicines12061240

**Published:** 2024-06-03

**Authors:** Dario Troise, Barbara Infante, Silvia Mercuri, Valeria Catalano, Elena Ranieri, Giovanni Stallone

**Affiliations:** 1 Nephrology, Dialysis and Transplantation Unit, Advanced Research Center on Kidney Aging (A.R.K.A.), Department of Medical and Surgical Sciences, University of Foggia, 71122 Foggia, Italy; 2 Renal Medicine and Baxter Novum, Department of Clinical Science, Intervention and Technology, Karolinska Institutet, 141 52 Stockholm, Sweden; 3 Unit of Clinical Pathology, Advanced Research Center on Kidney Aging (A.R.K.A.), Department of Medical and Surgical Sciences, University of Foggia, 71122 Foggia, Italy

**Keywords:** dendritic cells, immunotolerance, cancer, posttransplant malignancies, transplantation, immunosuppressive therapy

## Abstract

Dendritic cells (DCs) are a heterogeneous group of antigen-presenting cells crucial for fostering allograft tolerance while simultaneously supporting host defense against infections and cancer. Within the tumor microenvironment, DCs can either mount an immune response against cancer cells or foster immunotolerance, presenting a dual role. In immunocompromised individuals, posttransplant malignancies pose a significant health concern, with DCs serving as vital players in immune responses against cancer cells. Both recipient- and donor-derived DCs play a critical role in the rejection process, infiltrating the transplanted organ and sustaining T-cell responses. The use of immunosuppressive drugs represents the predominant approach to control this immunological barrier in transplanted organs. Evidence has shed light on the immunopharmacology of these drugs and novel strategies for manipulating DCs to promote allograft survival. Therefore, comprehending the mechanisms underlying this intricate microenvironment and the effects of immunosuppressive therapy on DCs is crucial for developing targeted therapies to reduce graft failure rates. This review will delve into the fundamental immunobiology of DCs and provide a detailed exploration of their clinical significance concerning alloimmune responses and posttransplant malignancies.

## 1. Introduction

Dendritic cells (DCs) are derived from hematologic precursors and represent a heterogeneous population of antigen-presenting cells that are crucial in supporting host protection against pathologic infections and cancer development/progression. They also normally play seminal roles in the generation of self-tolerance and prevention of autoimmunity, mediated by antibodies and immune effector cells [1].

In the “physiologically artificial” environment of allograft recipients, DCs appear to be critically involved in the mutual regulation of immune responsiveness between recipient and donor leukocytes, determining the balance between microchimeric tolerance and pathologic graft-versus-host/host-versus-graft immunity [2]. In such cases, where the administration of immunosuppressive agents is necessitated to limit DC alloreactive T-cell reactivity [3], it is perhaps not surprising that the natural protective functions of these immune cells are also subverted, resulting in elevated rates of infection and cancer development in drug-treated transplant recipients. This review will describe basic DC immunobiology and provide more in-depth insight into the clinical relevance of these antigen-presenting cells with regard to alloimmune responses and posttransplant malignancy.

## 2. Dendritic Cell Differentiation and Subtypes

Dendritic cells represent a remarkably heterogeneous population of sentinel cells that are distributed throughout the body’s tissues [4,5]. They can be categorized into various subsets based on their anatomic location, phenotype, and functional attributes. At least four stages of DC development have been described: bone marrow progenitors; precursor DCs, which are distributed throughout distal tissues and, upon pathogen recognition, release large amounts of cytokines, limiting the spread of infection; tissue-resident immature DCs, which possess a high degree of phagocytic capacity that permits the capture and processing of local antigens and the consequent transport of these immunogenic substrates to tissue-draining lymph nodes; and mature DCs that have been trafficked to secondary lymphoid organs where they are competent to optimally activate antigen-specific T cells and B cells. 

Dendritic cell progenitors are continuously produced from hematopoietic stem cells within the bone marrow [6]. CD34^+^ hematopoietic stem cells differentiate into common myeloid progenitors (CMPs) and common lymphoid progenitors (CLPs). Subsequently, CD34^+^ CMPs differentiate into CD11c^+^CD1a^+^ and CD11c^+^CD1a^-^ immature DCs. CD11c^+^CD1a^+^ immature DCs migrate into the skin epidermis and differentiate into Langerhans cells (LCs), whereas CD11c^+^CD1a^-^ immature DC cells migrate into the skin dermis and other tissues, where they differentiate into interstitial DCs (IDCs) [7]. In the absence of antigenic/pathogen stimulation, both LCs and IDCs may undergo steady-state migration to the draining lymph nodes, where they may play a critical role in supporting peripheral tolerance to normal “self” antigens due to their capacity to stimulate regulatory T cells. Upon microbial invasion and/or inflammation, tissue LCs and IDCs become activated by so-called “danger signals” and rapidly migrate into lymph nodes where they may prime or reactivate specific, protective B-cell and T-cell responses. Notably, LCs and IDCs differ in their capacity to activate different lymphocyte subsets, with IDCs preferentially inducing the differentiation of naive B cells into immunoglobulin-secreting plasma cells and LCs being particularly efficient activators of CTLs that mediate dominant antiviral and antitumor functions [8]. In addition to these two subsets of immature DC cells, CMPs also give rise to myeloid DC precursors (monocyte-derived pre-DC1), and CLPs give rise to plasmacytoid DC precursors (pre-DC2). These two DC types display many phenotypic differences [9]. Myeloid DC1 exhibits a CD11c^+^/CD123^dim^/BDCA-2^-^ phenotype and is also CD4^lo^ and CD33^dim^, with the ability to phagocytize antigens in immune complexes, given their expression of Fc receptors (CD32, CD64, FcΣRI). DC1 produces large amounts of IL-12 and induces strong T helper type 1 (Th1) and cytotoxic T-lymphocyte (CTL) responses. The CD11c^+^/CD123^dim^ myeloid DC1 migrates to tissues where, as immature DCs, they encounter and rapidly take up antigen. In the absence of infection or inflammation, immature DCs reside in the tissues for 4–5 days before migrating to the regional lymph nodes. In contrast, in the presence of danger signals and maturation stimuli, DCs are stimulated to rapidly migrate to the regional nodes and alter their phenotype to favor antigen presentation. In contrast, CD11c^-^/CD123^hi^/BDCA-2^-^ “plasmacytoid” DC2 cells express neither myeloid lineage marker (CD13, CD33) nor Fc receptors, and they are poorly phagocytic compared with myeloid DC. Because CD123 expression can also be upregulated on myeloid DC1 in culture, making the distinction between myeloid and plasmacytoid DC somewhat difficult, BDCA-2 may be preferred as a differential marker of DC2 (as this is not expressed by DC1 and appears to correspond to the CD123^hi^ cohort of DC2). DC2 cells are poor producers of IL-12 and tend to promote Th2-type responses associated with humoral immunity or the generation of IL-10-producing CD8^+^ T-suppressor cells. DC2 cells are impressive natural producers of type 1 interferon (IFN)-α, which represents a key effector molecule in protective antiviral (innate) immune responses [10].

While early studies attempted to support a simplistic linkage between DC1 and T-cell-mediated immune potentiation and DC2 with immune tolerance/suppression, we now appreciate that such correlations are not completely true and that the microenvironment present during DC generation/activation strongly influences whether T-cell activation occurs and what type of functional polarization is assumed by responder T-effector cells. For instance, after exposure to inflammatory cytokines (tumor necrosis factor [TNF]-α IFN-γ, IL-1), human DC2 can become functionally DC1-like in promoting type 1 (i.e., IFN-γ secreting) T cells. Interestingly, Itai et al. analyzed the transcriptomes of almost 130,000 CD8^+^ T cells from tumor-bearing mice following immune checkpoint blocking monoclonal antibodies therapy, finding an increased expression of cDC1 chemoattractant associated with CD8^+^ cytotoxic responses in the tumor microenvironment (TME) [11]. Therefore, further studies are needed to define this complex link. Nowadays, we can classify DCs as steady-state DCs, LCs, and inflammatory DCs. Steady-state DCs can be further classified into conventional DCs 1 and 2 (cDC1 and cDC2) and plasmacytoid DCs (pDCs) [12].

In the human spleen, tonsils, and lymph nodes, there appear to be several different DC subsets (at least five) that occupy anatomically distinct sites [13,14]. Cells with DC-like qualities were identified in B-lymphocyte-rich follicles, follicular mantle zone, and the T-lymphocyte-rich zones of the periarteriolar lymphoid sheath. At least three DC subsets (categorized based on phenotype and cytokine production profiles) have also been described in the thymus, where they are the principal mediators of the negative selection of the T-lymphocyte repertoire [15].

Newly generated DCs migrate via the bloodstream to peripheral tissues where they reside as immature cells, with certain subsets exhibiting high phagocytic capacity. Moreover, following pathogen invasion or after exposure to inflammatory mediators, DCs undergo phenotypic and functional maturation [16]. Upon maturation, DCs in tissues (skin, non-lymphoid interstitial sites, and mucosal surfaces) migrate into the afferent lymphatic system and move to T-cell-rich areas of lymph nodes, where they encounter naive T cells and initiate adaptive immune responses. Chemokine receptors are important for regulating DC localization and homing. Immature DCs express CXCR1, CCR1, CCR2, and CCR5, which confer responsiveness to inflammatory chemokines, such as macrophage inflammatory protein (MIP)-1α, MIP-1β and regulated on activation, normal T expressed and secreted (RANTES), directing the migration of DCs toward inflammatory stimuli and peripheral tissues [17]. Different DC subsets display unique sensitivity to certain chemokines. For example, MIP-3 has little effect on immature monocyte-derived DCs but, instead, recruits LCs. MIP-3 expression is restricted to the activated epithelium, as observed in tonsils and gut, and its induction during inflammatory processes appears fundamental for the migration of immature LCs or their precursors to sites of inflamed epithelia. During such migration (i.e., after antigen exposure), LCs downregulate their level of cell surface E-cadherin, releasing tethers to the basement membrane and allowing these DCs to emigrate from the skin [18].

## 3. Dendritic Cell Maturation 

Numerous factors induce and/or regulate DC maturation in situ, including pathogen-related molecules, such as lipopolysaccharide, bacterial DNA, and double-stranded RNA, as well as the balance between proinflammatory and anti-inflammatory signals in the local microenvironment and T-cell-derived signals, including TNF, IL-1, IL-6, IL-10, transforming growth factor-β (TGFβ), and prostaglandins. Two families of receptors appear to be critical for DC activation: the microbe-responsive toll-like receptors (TLR) and TNF-family receptors, among which CD40 seems to be a particularly dominant player. It currently remains unclear whether bacterial products interact directly or indirectly with TLR, and, in some cases, endocytosis of the maturation stimulus may precede TLR activation (i.e., signals are generated within intracellular organelles). The involvement of TLR in maturation provides a mechanism through which DC effectively links innate to adaptive immunity [19,20]. After detecting microbial products or proinflammatory cytokines, immature DCs are induced to undergo phenotypic and functional changes that ultimately culminate in their conversion from an antigen-scavenging/capturing cell to a mature DC, competent to transport antigens to peripheral secondary lymphoid organs, where cognate T- and B-cell activation may occur. During this process, DCs undergo a multistage metamorphosis, in which they lose their capacity to take up exogenous antigens but acquire increased surface expression and stability of MHC class I and class II peptide complexes, as well as adhesion and co-stimulatory molecules, important for the activation of adaptive immunity (i.e., CD40, CD54, CD80, CD86), and these cells become competent to produce and secrete high levels of proinflammatory cytokines, such as IL-1, IL-6, IL-12, IL-18, and IL-23 [21]. Indeed, when comparing mature versus immature DCs, cell surface MHC class II complexes can increase some 5–20-fold, while CD86 co-stimulatory molecule expression may increase up to 100-fold. Interestingly, the increase in cell surface MHC class II complexes on mature DCs largely reflects posttranslational events, with preformed protein exported from intracellular stores (i.e., in lysosomes/endosomes) to the plasma membrane (and little change in MHC class II mRNA transcript levels) [17]. Morphologic changes accompanying DC maturation include a loss of adhesive structures involved in DC tethering within tissues, cytoskeleton reorganization, and the acquisition of high cellular motility. Most notably, maturing DCs extend long “dendritic” processes that increase their ability to “communicate” with other immune cells (i.e., other DCs, as well as B, T, and NK cells) that optimize their profound immunomodulatory functions [22]. Indeed, the maturation state of DCs (or the way they matured) is considered a key determinant in the outcome of T-cell activation, resulting in either T-cell tolerance or active immunity. Hence, the type of stimuli involved in DC maturation (i.e., activating DC via TLR vs. other signaling receptors) can dictate how and whether DCs activate polarized T-cell responses or whether functional T-cell tolerance occurs. Stimuli derived from pathogens (lipopolysaccharide, bacterial CpG DNA, and double-stranded viral RNA), as well as T-cell signals (CD40 ligand and IFNy), promote immature DCs to produce IL-12 and prime for Th1 responses. In contrast, anti-inflammatory molecules, such as IL-10, TGFβ, prostaglandin E2 (PGE_2_), and corticosteroids, inhibit DC maturation and IL-12 production, resulting in predominantly Th2 or regulatory T-cell responses associated with tolerance [23,24,25].

## 4. Dendritic Cell Antigen Uptake Processing and Presentation

Although DCs comprise multiple subsets, many of these are remarkably effective at antigen processing and presentation during their immature state of differentiation [26]. Antigens can be avidly taken up by immature DCs via diverse mechanisms, including macropinocytosis [27], receptor-mediated endocytosis via C-type lectin receptors (mannose receptor [CD206] and DEC-205 [CD205]) or Fc receptor types I (CD64) and (CD32) [28,29], and phagocytosis of particles, such as latex beads, apoptotic, and necrotic cell fragments (involving CD36 and the α_v_β_3_ or α_v_β_5_ integrins), viruses, and bacteria [30]. Dendritic cells can also internalize exogenous antigens complexed with heat shock proteins such as gp96 and Hsp70 (as released from stressed/dying cells) through scavenging receptors [31]. Interestingly, the expression of mannose receptors differs among the various DC subsets, yielding differential competency to acquire glycosylated antigens. Indeed, circulating CD11c^+^ DCs and LCs do not express these receptors, in contrast to IDCs and in vitro generated monocyte-derived DC1. On the other hand, LCs but not IDCs express a novel C-type lectin (called Langerin-CD207) that may play a differential role in the preferential uptake and processing of certain antigens and the consequent unique ability of LCs to activate T-effector cells, exhibiting a unique repertoire relevant to host protection against infectious disease [32,33]. The uptake of exogenous antigens by receptor-mediated endocytosis, phagocytosis, or macropinocytosis leads to antigen processing through the major histocompatibility complex (MHC) class II pathway [34]. Antigens are degraded in endosomes, and the generated polypeptides are transported into MHC class II-rich compartments for their loading onto nascent MHC class II molecules. In immature DCs, abundant MHC class II molecules are synthesized, but they are mainly sequestered intracellularly in late endocytic compartments (lysosomes) [35]. The antigens captured are targeted to MHC class II-positive lysosomes. However, they are not efficiently used for the formation of MHC II-peptide complexes but are retained for use as immunogenic peptides days later. Immature cells form stable class II dimers but do so without generating immunogenic complexes. Thus, immature DCs in culture can take up antigens but do not present them efficiently to T cells. After detecting microbial products or proinflammatory cytokines, immature DCs transform into mature DCs. This transition is accompanied by dramatic cytoplasmic reorganization, highlighted by a redistribution of MHC class II from intracellular compartments to the plasma membrane. Class II molecules appear to exit the lysosomes, then reside transiently in non-lysosomal cytoplasmic structures (class II vesicles), and finally accumulate on the cell surface. One element of the mechanism, possibly unique to DC, may involve the regulation of cathepsin-S activity by the specific anti-protease cystatin [36]. Cathepsin-S plays a major role in the cleavage of the MHC Il-associated invariant (Ii) chain, which is inhibited by cystatin C in immature DC. After maturation, cystatin C is downregulated, and the activity of cathepsin S increases, promoting li degradation and allowing for the export of peptide-loaded class II molecules to the cell surface [37]. In contrast to exogenous antigens, endogenously synthesized antigens of viral or cellular origin are typically processed by the proteasome in the cellular cytoplasm. Peptide fragments generated by the proteasome may be transferred into the endoplasmic reticulum by the (adenosine triphosphate-dependent) transport-associated protein (TAP-1/TAP-2) complex, where they may bind to nascent MHC class I molecules. Appropriately folded MHC class peptide complexes become transport-competent and are then shuttled to the cell surface for presentation to CTL [38]. For certain cell types, and particularly for certain DC subsets, the demarcation between endogenous and exogenous antigen presentation is not very strict, and the MHC class I-restricted presentation of both intracellular and exogenous sources of antigen can occur efficiently. The unusual capacity to direct exogenous antigens into the MHC class I antigen processing pathway is termed “cross-presentation”, and the subsequent initiation of a CD8 T-cell response by DCs performing this function is called “cross-priming”. The mechanisms underlying cross-presentation have only recently begun to become clear. Evidence suggests that cross-presentation may involve the fusion of the endoplasmic reticulum with early phagosomes to form organelles that contain all the requisite components of the MHC class I-processing machinery. Phagocytosed proteinaceous antigens may be retro-translocated out of the phagosome and into the cytoplasm, where they are degraded by proximal proteasomes. Fragmented peptides may then be transported back into the phagosome by the TAP complex and loaded onto newly formed or even recycled MHC class I molecules for subsequent transport to the cell surface [39]. Several types of antigens have been reported to be cross-presented by DC. These include soluble proteins, such as immune complexes, intracellular bacteria, parasites, and cellular antigens. Indeed, at least certain DC subsets are competent to cross-present tumor antigens acquired from phagocytosed tumor cells for presentation to antitumor and antiviral CD4+ and CDS’ T cells. In this regard, recent reports have reemphasized the importance of (antigen-specific) CD4^+^ T-cell “help” in amplifying and sustaining protective CD8^+^ T-cell-mediated responses [40], thus underscoring the physiologic benefits for coordinating the processing and presentation of antigens via both the MHC class I and II pathways.

## 5. Dendritic Cell/T-Cell Interactions and Immunological Outcomes

To become activated, cognate T cells need to establish a so-called “immunologic synapse” with antigen-loaded DCs, in which the T-cell receptor (TcR) and co-stimulatory/co-inhibitory molecules are congregated in a central area surrounded by a ring of adhesion molecules on the T-cell surface at the site of interaction with DC. At the synapse, serial TcR triggering by binding to DC-expressing MHC–peptide complexes initiates a signaling cascade. If the magnitude and duration of the net balance of signals exceed a critical threshold potential, naive T cells may enter the cell cycle and differentiate into effector T cells. If this threshold is not attained, functional anergy may occur, with T cells undergoing limited rounds of replication without acquiring effector cell status [41]. The interaction between the co-stimulatory molecules CD80 and CD86 expressed on DCs with their receptors CD28 and CTLA-4, expressed on T lymphocytes, may provide a means by which immunity and tolerance are balanced. In the absence of inflammation, CD80/86 expression on DCs is low, and their high-affinity interaction with CTLA-4 on regulatory T cells (Tregs) is dominant, maintaining tolerance. In contrast, in inflamed tissue, CD80/86 expression is higher on activated DCs, allowing for improved stimulatory engagement of CD28, resulting in an aggregate milieu that is more conducive to antigen-specific effector T-cell priming [42]. In addition, a range of co-inhibitory molecules (i.e., PD-L1 and B7-H4, among others) have been recently defined on DC that can “antagonize” the positive influence of co-stimulatory interactions, resulting in the down-modulation or negation of T-cell priming [43]. Moreover, DC/T-cell interaction results in the reciprocal stimulation of both cell types and a temporal dialog between these two cells. For instance, only after the initial T-cell interaction is the molecule CD40L (CD154) expressed on the T-cell surface, allowing for its interaction with constitutively expressed CD40 on DC. This interaction promotes the production of cytokines from DC, such as IL-12, which mediates the potent co-stimulation of polarized type 1 (i.e., IFNy-associated) CD4^+^ and CD8^+^ T-cell immunity. Given the kinetics of such a dialog, it is inherently obvious that the duration of TcR stimulation and the degree of advancement of the DC/T-cell dialog have profound implications regarding the degree and nature of the resulting differentiation and durability of effector T cells. Factors (i.e., inflammation, anti-inflammatory agents, tumor microenvironment, age) that may condition or alter the profile of (DC-produced) cytokines available for differentiating T cells also clearly impact the numbers and functional competency of responder T cells [44]. In addition, one must consider whether different DC subsets are endowed with unique capacities to induce Th1, Th2, or Treg responses. In humans, monocyte-derived CD11c^+^ DCs predominantly polarize naive T cells toward a Th1 profile [45], whereas the CD11c^-^ DC subset induces T cells to predominantly produce Th2 cytokines [46]. The extent of T-cell polarization by CD11c^-^ DCs may be related to their differentiation/maturation stages. The mechanisms by which CD11c^-^ DC induce Th2 cytokines have not been conclusively determine but likely involve the differential impacts of IL-6, IL-13, and OX40L [47]. Human plasmacytoid DCs appear to be well suited to promote T-cell hyporesponsiveness or active T-cell tolerance (mediated by Treg). Thus, a given T-cell-mediated response to an antigenic challenge is dependent on a spectrum of factors, including the type and maturity of the priming DC subset, the durability of the DC/T-cell interaction, the balance of co-stimulatory versus co-inhibitory T signals provided in the context of antigenic stimulation, and the cytokine milieu, with profound effects evidenced at the level of T-cell tolerance, immunity, and memory.

## 6. Dendritic Cells and Cancer 

DCs play crucial, and likely ever-changing, roles in cancer patients. There is a distinct milieu, where hypoxia, lactate accumulation, and the infiltration of a substantial number of immunosuppressive cells are features that distinguish the TME from normal tissues [48,49]. The role of the immune system in the TME is crucial. The accumulation of cells with immunoregulatory functions, including regulatory T cells (Tregs), myeloid-derived suppressor cells (MDSCs), and tumor-associated macrophages (TAMs), is one of the most notable characteristics of the TME [50]. Interestingly, immune cells represent two faces of the same coin because they can either promote tumorigenesis or suppress tumor development. Depending on the context and by providing environmental signals, the TME can influence the fate of DCs by inducing an immune response to cancer cells or promoting immunotolerance [51]. DCs are required for recruiting and priming effective T-cell responses and for the maintenance of effector memory T-cell function in the TME, as shown by several preclinical models [52,53,54].

**Figure 1:**
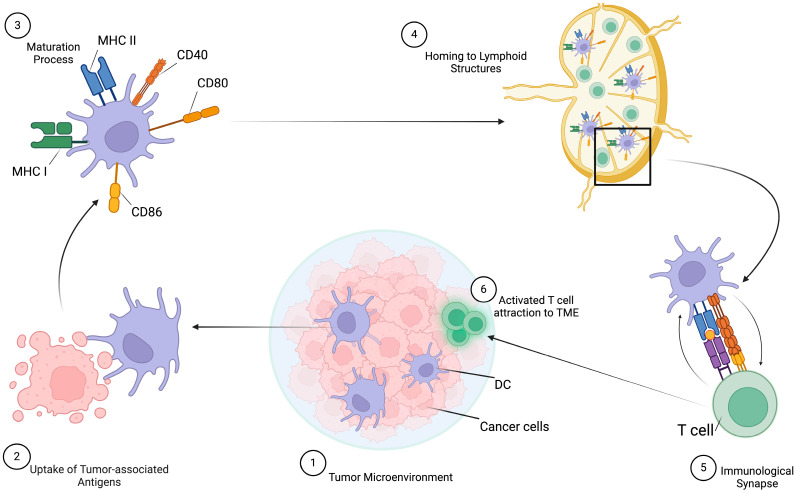
Dendritic cells and anti-neoplastic response. The anti-neoplastic immune response initiated by intra-tumoral DCs relies on a sequential process, beginning with the recognition, uptake, and processing of tumor-associated antigens. Subsequently, DCs undergo a maturation process characterized by the upregulation of MHC class I and class II molecules, increased expression of costimulatory molecules, such as CD40, CD80, CD86, and, finally, their homing to lymph nodes or lymphoid structures to encounter and activate T cells. This process will result in the eradication of tumor cells. Created with BioRender.com.

The anti-neoplastic response depends on a step-by-step process, and DCs play a pivotal role in T-cell activation and immune response initiation. In summary, tumor-associated antigens (TAAs) released from apoptotic or necrotic tumor cells are recognized, phagocytized, and processed by DCs in immunogenic peptides that are loaded onto major histocompatibility complex (MHC) class I or class II molecules for presentation to CD8 T cells and CD4 T cells, respectively. Afterwards, DCs require a maturation process, which includes the upregulation of MHC class I and class II molecules, increased expression of costimulatory molecules such as CD40, CD80, and CD86, and secretion of cytokines in order to enhance T-cell stimulation. Moreover, there is an upregulation of CC-chemokine receptor 7 (CCR7) to improve DC homing to tumor-draining lymph nodes or to tertiary lymphoid structures via lymphatic vessels or blood circulation, where they encounter and activate T cells. To facilitate T-cell priming, this maturation process is essential; in the absence of DC activation, tolerance and immune regulatory responses are promoted [55]. (Figure 1).

Tumor-derived TGF-β hinders the migration of dendritic cells (DCs) to tumor-draining lymph nodes through both paracrine and autocrine mechanisms. For example, increased levels of TGF-β in the TME can lead to higher levels of vascular endothelial growth factor (VEGF), which impair DCs functions by inhibiting their differentiation from precursors, activation, and recruitment to the tumor site in a paracrine way [56]. Moreover, reports have indicated that TGF-β produced by the tumor can travel to the tumor-draining lymph node and induce apoptosis in DCs [57]. The autocrine effect of TGFβ has also been demonstrated to maintain the activation of indoleamine 2,3-dioxygenase (IDO) in DCs and preserve their tolerogenic functions. Furthermore, TGF-β can directly inhibit CCR7 expression on DCs, reducing the migratory response to lymphoid chemokines [58].

In skin disorders, LCs play a crucial role in immune system regulation, with some of their immunomodulatory effects attributed to the production of TGF-β. Bobr et al. showed that the removal of TGF-β or its receptor from LCs causes significant migration of these cells to the regional lymph node, both under normal conditions and during inflammation [59]. Moreover, in skin tumor models, TGF-β prevents tumor infiltration and LC migration to skin-draining lymph nodes, facilitating tumor evasion from the immune system [60]. Tumor cells have developed several strategies to prevent recognition by DCs. One such strategy involves tumor-derived stanniocalcin-1 (STC1) interacting with calreticulin (CRT), one of the best identified damage-associated molecular patterns (DAMPS), which takes part in dead tumor cell removal because it can act as a “receptor”. This interaction prevents CRT from being exposed to the tumor cell membrane and, consequently, inhibits the phagocytosis of tumor cells by DCs. An elevated expression of STC1 in tumors is closely associated with reduced responses to immunotherapy in patients [61]. Moreover, the expression of TIM-3 (T-cell immunoglobulin- and mucin-domain-containing molecule-3), initially identified as a marker of T-cell exhaustion, has been observed in other leukocyte populations, including macrophages, natural killers, and DCs. TIM-3 is a type I trans-membrane protein, and its primary ligands include Galectin-9, the most studied ligand and crucial regulator of tumor cell immune evasion. The interaction of TIM-3/Galectin-9 has been shown to induce CD8^+^ T-cell death in colon cancer. Other ligands are high-mobility group box 1 (HMGB1) and carcinoembryonic antigen cell adhesion molecule 1 (Ceacam1), both implicated in the regulation of immunoresponses, and phosphatidylserine (PtdSer), implicated in apoptotic cell uptake. Among these interactions, the Tim-3/Galectin-9 and TIM-3/Ceacam1 interactions result in similar downstream events because they bind to different regions in the IgV domain of Tim-3 and predominate in tumor cell immune escape mechanisms [62].

Notably, a high expression of TIM-3 was detected in exhausted T cells during graft-versus-host disease, chronic infection, and cancer, but its expression was also found in intratumoral CD103^+^ DCs. The use of the anti-TIM-3 antibody stimulates the expression of CXCL9, leading to enhanced functions of CD8^+^ T cells and improving the therapeutic activity of paclitaxel in a breast cancer model. Therefore, TIM-3 could play a pivotal role in tumor cell immune escape mechanisms [63]. Bagdadi et al. showed that the expression of TIM-4, similar to TIM-3, on tumor-associated macrophages and DCs hinders tumor antigen presentation by activating autophagy [64]. The secretion of factors produced by tumor cells plays a pivotal role in inducing DC tolerance. These factors, PGE_2_, TGF-β, and VEGF, subsequently induce the production of other immune-modulating substances. Tumor-derived PGE_2_ suppresses the activation of DCs by reducing IL-12 production, inhibiting co-stimulation and upregulating programmed death-ligand 1 (PD-L1) and arginase 1 (Arg1). Furthermore, tumor-derived TGF-β impairs the ability of pDCs to react to innate immune signals and inhibits type I IFN secretion, contributing to tumor growth by suppressing the infiltration of natural killers and recruitment of regulatory T cells [65]. VEGF is considered a major cytokine in the TME because most cancer cells secrete it as an angiogenic factor to promote endothelial cell proliferation. VEGF impairs the motility and immune function of DCs through VEGF-receptor 2 and upregulation of RhoA-GTPase. This suggests that the inhibition of mature DC motility by VEGF serves as one of the immune evasion tactics of cancer cells [66]. In addition, tumor cells, tumor-associated macrophages, and Treg cells often produce IL-10 or shed gangliosides, which may also preclude optimal DC maturation and function. Such immature or partially differentiated myeloid DCs are anticipated to induce either inappropriate (type 2-, Treg-) T-cell responses or to yield anergy/tolerance rather than potent antitumoral T-cell-mediated immunity associated with stimulation by mature DC1 in vivo [67,68]. Tregs are one of the major cells capable of inducing dysfunction in DCs through the expression of high-affinity CD80 and CD86 ligands, cytotoxic T-lymphocyte-associated antigen 4 (CTLA-4), which competes with CD28 on T cells and limits tumor immunogenesis [69]. The existence of DCs in tumors resistant to immunotherapy suggests possible alterations in the functions of these cells within specific neoplastic lesions. Using single-cell RNA sequencing in both mouse and human non-small-cell lung cancer models, Maier et al. identified a unique subset of DCs named “mature DCs enriched in immunoregulatory molecules” (mregDCs). These cells are distinguished by the simultaneous expression of regulatory genes and maturation markers [70]. MregDCs have been documented in several human cancers, such as head and neck lymphoma, hepatocellular carcinoma, gastric cancer, colon cancer, and breast cancer. In particular, the examination of tumors and metastatic lymph nodes from patients diagnosed with head and neck lymphoma indicates that mregDCs might influence the prognosis by regulating the balance between regulatory and effector T cells [71].

Cells of all tissues, including neoplastic and immune cells, are able to release extracellular vesicles (EVs), which are formed of a double layer of lipid membranes. EVs play a role in regulating homeostatic processes and contribute to the development and progression of various diseases, including cancer. Three types of EVs are known: exosomes originate as intraluminal vesicles and are then released through fusion with the plasma membrane; microvesicles are generated by direct budding from the plasma membrane of living cells; and apoptotic bodies are released from apoptotic cells. Two functions have been observed involving the interaction between tumor EVs and DCs: they can either enhance or suppress antigen presentation. For example, EVs from melanoma were found to impede the differentiation of monocyte-derived DCs in vitro, inducing a myeloid-derived suppressor cell phenotype that reduces the antitumor function of DCs. In an animal model of ovarian carcinoma, EVs were found to transport Arg1. This molecule depletes L-arginine, which is crucial for antigen presentation by DCs and for T-cell expansion. Contrarily, there is evidence indicating that tumor cells may use DC cross-dressing to transfer MHC/antigen complexes to DCs, using EVs as carriers to deliver antigens and immunostimulatory molecules [72].

Ongoing research is required to face the risks and challenges when it comes to optimizing the application of DCs in cancer immunotherapy and enhancing their safety and efficacy profiles. Cancer cells can develop mechanisms to evade immune responses mediated by DCs. One of the mechanisms is the production of cytokines, such as IL-6, VEGF, and IL-10, which suppress dendritic cells via STAT3 signaling. Tumors may also condition local DCs to generate suppressive T cells, such as FOXP3^+^ T cells. Moreover, studies showed that in multiple myeloma, DCs can even support the clonogenic growth of tumors [73]. Some evidence reported that immunotherapy with DCs could lead to severe autoimmune disease when the cancer antigens are not tumor-specific but are also expressed in peripheral nonlymphoid organs [74].

Despite these challenges, DCs can be considered the cornerstone of immune responses against tumors because they are capable of modulating T-cell responses. Although the presence of DCs within tumor lesions has been typically correlated with improved clinical outcomes, a more comprehensive determination of intralesional DC function and how this varies with disease stage or time since diagnosis (as an index for the period of immune deviation that might be associated with tumor presence), or how such DC infiltrates correlate with circulating levels of tumor-specific T cells, has not yet been made. Thus, Tregs are emerging as a critical cell subpopulation in the TME that acts directly on cells inducing DC dysfunction, and their depletion may offer potential benefits for the mediation of DC antitumor immunity.

## 7. Dendritic Cells and Transplantation

Over the past five decades, organ transplantation has witnessed significant expansion, with an increasing number of patients with end-organ failure benefiting from kidney, liver, heart, and lung transplantation. Advances in surgical techniques, ancillary care, and the widespread adoption of multidrug immunosuppressive regimens have resulted in markedly improved short-term (1–3 year) patient and graft survival rates. For many organ systems, such as the kidney, intestine, liver, pancreas, heart, and lung, the one-year graft survival rates now approach or exceed 80%. However, despite these advancements in short-term graft survival and a notable reduction in acute rejection rates, there has been limited progress in addressing long-term graft attrition [75]. Although there are clearly nonimmune factors that contribute to late graft loss (i.e., infection, drug-specific toxicities, hypertension, dyslipidemia), alloimmunity plays a dominant role in the chronic rejection of most transplanted organs (i.e., kidney, heart, lung), with the activation of such harmful (largely T-cell-mediated) immunity being strongly dependent on the function of stimulatory (donor as well as recipient) DCs. The majority of recipient DC cells that replace donor DCs in heart and kidney grafts originated from non-classical monocytes and were characterized by IL-12 production and CD11b^+^ and CD11c^+^ markers, such as the one present in sterile inflammation compared to the ones found in infection models, where most DCs produce TNF. Moreover, Th1 represents the principal T-cell phenotype profile that can be selectively promoted by DCs during T-cell differentiation. Thus, organ transplantation represents a unique model for the induction of a monocyte-specific differentiation program, in which allogenic stimuli are combined with sterile inflammation to elicit an immune response against the transplanted organ [76]. Using an immunoimaging approach, Garrod et al. showed that natural killer (NK) cells scan the lymph nodes and eliminate donor-derived DCs within hours after transplantation. To better define the mechanisms underlying alloantigen presentation, they ablated CD11c^+^ DCs, resulting in prolonged graft survival, suggesting that the immune response to the transplanted organ is impaired by the absence of DCs [77].

**Figure 2:**
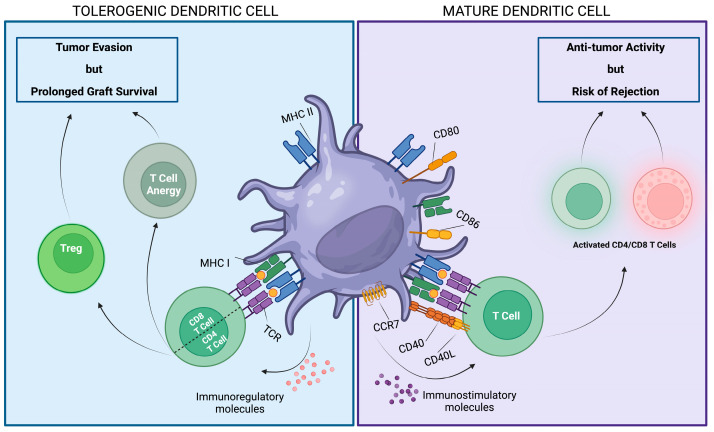
Dendritic cells: two faces of the same coin. DCs play a pivotal role in allograft tolerance and in anti-neoplastic response. Mature DCs are distinguished by elevated expression levels of MHC class I/II and immunostimulatory molecules showing the ability to activate CD4/CD8 T cells exerting antitumor activity or graft rejection, depending on the context. Conversely, Tol-DCs are typically characterized by diminished expression of costimulatory molecules and heightened levels of immunoregulatory molecules associated with the development of anergic T cells and increasing number of Treg cells, which can lead to tumor evasion or tolerance of the graft. Created with BioRender.com.

Recipient- and donor-derived DCs play a critical role in rejection by infiltrating the transplanted organ and maintaining T-cell responses and also play a pivotal role in allograft tolerance. While mature DCs are characterized by high expression levels of costimulatory molecules and class II MHC, DCs that exhibit the ability to suppress immune responses are called tolerogenic DCs (Tol-DCs) and are often identified by a lower expression of costimulatory molecules and increased expression of anti-inflammatory molecules, including IL-10 and TGB-β [78,79] (Figure 2).

In the liver, cDCs are predominantly located in the periportal and pericentral space, and after transplantation, they undergo gradual maturation under the influence of inflammatory mediators, such as TNF-α, IFN-β, -γ, IL-6, and PGE_2_. Ischemia–reperfusion injury (IRI) is considered a critical factor involved in donor DC maturation and migration to recipient secondary lymphoid tissues. Additionally, Lutz et al. proposed that full DC maturation is required for the generation of T-cell responses, whereas Tol-DCs may be in a semi-mature state, inducing Tregs, T-cell weakness, and promoting T-cell apoptosis [80]. Tol-DC in the liver may be promoted by hepatic stellate cells, which are considered the major source of immunoregulatory molecules, including all trans retinoic acid (ATRA), chemoattractants, such as MCP-1 and MIP-1α, as well as cytokines such as IL-6 and TNF-α. These molecules are involved in the upregulation of IDO, which leads to local tryptophan deficiency and subsequent inhibition of effector T cells. Moreover, ATRA participates in the induction of arginase 1 and nitric oxide secretion, both of which are associated with the inhibition of T-cell responses [81,82]. Hepatic DCs exhibit lower endocytic capacity and lower expression of class II MHC, which results in the reduced stimulation of T cells. Moreover, they showed the ability to inhibit CD8^+^ T cells because of the expression of higher levels of PD-L1 and reduced capacity to activate allogeneic naïve T cells. In contrast to DCs derived from extrahepatic tissues, under homeostatic conditions, hepatic DCs show a tolerogenic profile after liver transplantation in animal and human liver transplantation models [83,84].

pDCs are considered to play a role in liver graft rejection and tolerance, although they are not as effective as cDCs. They can be considered hybrid cells because they share some features of both classical DCs and lymphocytes due to the rapid and massive production of type I IFN and their antigen presentation ability. Furthermore, they show the capacity to produce costimulatory molecules and express class II MHC molecules, which allow them to present antigens to CD4^+^ T cells and regulate the viability of NK cells and macrophages [85]. On the other hand, they also drive immunotolerance, supporting the development of natural Tregs and promoting allogeneic CD4^+^ T-cell apoptosis due to the lower expression of the Delta4/Jagged 1 Notch ligand ratio, which is involved in the Th2 cell differentiation process [86].

Regarding cardiac transplantation, after the first year after transplantation, rising and detectable graft infiltration of DCs has been found. It has been hypothesized that lymphatic disruption may occur after heart transplantation, leading to a reverse transmigration of DCs into the blood circulation to present donor antigens to alloreactive T cells. The activation and stimulation of T cells occur, at least partially, at the level of the transplanted heart, as suggested by Kofler et al., who showed clusters of DCs with CD3^+^ T cells in myocardial graft biopsies [87]. In another report, CD11c^+^ DCs appeared to play a pivotal role in alloimmune responses against old cardiac allografts via IL-17A. Oberhuber et al. cocultured DCs from young and old mice with naïve CD4^+^ cells and showed that increased T-cell proliferation and IL-17A production are driven by DCs, leading to more potent alloimmune responses in older transplanted hearts and accelerating rejection. Increased survival of the allograft and reduced immunoresponses were achieved by the neutralization of IL-17A and depletion of donor DCs before surgery [88]. In an animal model, pDCs demonstrated a tolerogenic profile in vascularized cardiac grafts as they function as antigen-presenting cells (APCs), migrate to the lymph nodes, and induce the antigen-specific development of CD4^+^CD25^+^Foxp3^+^ Treg cells, thus prolonging graft survival [89].

In kidney grafts, DCs have been shown to be involved in the harmful activation of the immune system during IRI, but they also exert protective renal effects, depending on their maturity status. Thus, immature kidney DCs demonstrated an inhibitory effect on inflammation during IRI compared with mature kidney DCs. Moreover, IRI drives donor DCs to induce acute kidney rejection by presenting alloantigen to recipient T cells directly. In contrast, chronic kidney rejection is more likely mediated by recipient DCs due to their longer lifespan. Studies show that most donor DCs are replaced by recipient DCs within 24h after kidney transplantation. Current studies claim that hypoxia exposure during IRI influences DC quality and the intensity of immunoreaction by upregulating hypoxia-inducible factor (HIF). HIF-dependent changes affect cell maturation, survival, migration of DCs, and T-cell activation. Regarding the tolerogenic effects of DCs, systemic administration of donor-derived Tol-DCs induced T-cell hyporesponsiveness to alloantigens and prolonged kidney graft survival in nonhuman primates. Importantly, Tol-DCs did not induce the production of specific antibodies after their injection [90,91,92].

The predominant approach to override this immunologic barrier in organ transplantation is the application of immunosuppressive drugs that limit or preclude DC/T-cell function and subsequently prevent allograft rejection. Recently, an increasing number of studies have highlighted that several well-established immunosuppressive medications not only target the effector cells but also modulate key functions of DCs [93]. 

One of the most effective anti-inflammatory drugs with immunosuppressive properties is corticosteroids (CS), such as dexamethasone or prednisolone. Because of their characteristics, CS have been used for the treatment of autoimmune and inflammatory diseases as well as in immunosuppressive protocols. They are typically used with other immunosuppressive drugs to minimize side effects. While the therapeutic effects of CS were initially ascribed to the strong inhibitory effect on T cells, it is clear that antigen-presenting cells, including DCs, are also negatively affected by CS [94]. These agents inhibit DC maturation as well as the production of T-cell stimulatory cytokines in response to a range of activating stimuli. CS have been shown to reduce the production of inflammatory cytokines, including TNF-α and IL-1β, and to downregulate costimulatory molecules, such as CD40, CD80, CD83, CD86, and class II MHC, in DCs in response to CD40L and lipopolysaccharide [95,96]. In a mouse cardiac transplantation model, Tim et al. showed the upregulation of CD85k, a biomarker expressed on Tol-DCs, after treatment with a combination of CS and thalidomide, an anti-angiogenic and anti-neoplastic drug [97]. To improve organ transplantation outcomes, the latest research has focused on the development of cellular therapies using Tol-DCs and immunosuppressive medications. Additionally, recent evidence has provided new perspectives on the immunopharmacology of these compounds and novel strategies for DC manipulation to promote allograft survival [98]. Polymeric nanoparticles containing ovalbumin and dexamethasone may switch DCs into Tol-DCs, inducing antigen-specific immune tolerance, as shown by Kim et al. [99]. Another example is provided by Tol-DCs generated from dexamethasone and Vitamin D3, which are characterized by a decreased production of proinflammatory cytokines and a typical tolerogenic phenotype [100]. 

Calcineurin inhibitors (CNIs), such as cyclosporin (CsA) and FK506 (Tacrolimus), are potent and widely used immunosuppressive drugs that may mediate their effects, in part, via the targeted inhibition of costimulatory molecules and the allostimulatory capacity of both cDC and pDC subsets [101]. Ren et al. analyzed the phenotype and cytokine profile of DCs derived from human monocytes exposed to FK506 and found increased expressions of IL-10 and TGF-β after 24 h of tacrolimus stimulation. Moreover, FK506-treated DCs showed an inhibitory effect on CD4^+^ T-cell proliferation, suggesting a potential tolerogenic role of the cells when exposed to this immunosuppressive drug [102]. According to previous evidence highlighting the role played by calcineurin in the differentiation of DCs through the nuclear factor of activated T cell (NFAT), a transcription factor involved in the production of cytokines (e.g., IL-2) that promote T-cell proliferation, Colombo et al. showed that the inhibition of the NFAT signaling pathway with nanodrugs was capable of targeting phagocytes in vivo, suppressed Th1 cell responses, and led to an increased survival of the graft [103]. Moreover, in lung transplant patients, FK506 inhibits the maturation of DCs, impairing the antigen presentation to T cells and the subsequent adaptive immune responses [104].

Another class of immunosuppressive drugs is mTOR-inhibitors (mTORi). mTOR (mammalian target of rapamycin) is a serine/threonine kinase involved in the regulation of cell growth and metabolism. mTORi, including Rapamycin (RAPA) and Everolimus (RAD), are known as anti-neoplastic and immunosuppressive drugs and are used as anti-neoplastic drugs and in the transplantation field. Dahlqvist et al. characterized the effect of FK506 and RAPA on DCs and observed that RAPA-exposed DCs exhibited decreased expression of costimulatory markers and a more immature phenotype than DCs exposed to FK506 but reduced expression of pro-tolerogenic protein and cytokines, including IDO, PD-L1, and IL-10. In contrast, DCs exposed to FK506 showed an increased anti-inflammatory profile that could lead to acceptance of the graft but did not show phenotypical changes [101]. Moreover, the use of RAD has been associated with the expression of several immune checkpoint molecules. For example, increased mRNA levels of PD-L1, CTLA-4, V-domain Ig suppressor of T-cell activation (VISTA), and B- and T-lymphocyte attenuator (BTLA) were found in monocyte-derived DCs treated and incubated for 24 h with RAD. In terms of immunomodulatory effects, RAD has been shown to shift the DC phenotype toward a partially tolerogenic state [105]. Moreover, the use of RAPA in renal transplant recipients has been shown to trigger a rise in the number of pDCs, accompanied by a notable increase in Ig-like transcript 3 and 4 (ILT3–ILT4) expression levels, while simultaneously decreasing CD40 expression on their cell surface. ILT3 and ILT4 enhance pDC capacity to suppress the proliferation of allogeneic T cells. Thus, RAPA could potentially lead to a rise in circulating Tregs [106]. Klaeske et al. analyzed DCs derived from heart transplant recipients with mTORi- and CNI-based therapy to identify patients with an increased risk of rejection and observed higher levels of BDCA2^+^ and BDCA4^+^ pDCs and lower levels of BDCA1^+^ myeloid DCs in patients under an mTORi-based immunosuppressive regimen. They concluded that mTORi exerts an insufficient tolerance-promoting reaction in DCs, which could explain the increased risk of rejection in patients under mTORi-based therapy [107]. Mycophenolate mofetil (MMF) is a reversible inhibitor of inosine 5′-monophosphate dehydrogenase, which is a rate-limiting enzyme in the de novo synthesis of purines. Treatment of DCs with MMF suppresses the DC expression of co-stimulatory and adhesion molecules important for T-cell activation and limits DC production of IL-12 [108].

Nowadays, it is evident that targeting a single pathway is inadequate for most cancers, and novel molecules capable of modulating the immune responses by targeting specific interactions between DCs and T cells are being investigated. They are known as bispecific cell engagers (BiCE) and could have a potential role in potentiating antitumor immunity. For example, BiCE that promotes crosstalk between cDC1s and PD-1^+^ CD8^+^ T cells has been shown to induce a strong antitumor response in mouse models, including those resistant to traditional aPD-1 therapy. In addition, the lymphocytes in the TME shifted to a more proinflammatory phenotype, with reduced T-cell exhaustion and suppression activity [109]. Moreover, Lamendour et al. showed that the binding of two pathogen recognition receptors on the surface of DCs by BiCEs can switch the DC phenotype into a tolerogenic one, inducing Treg differentiation [110]. Thus, BiCE offers a unique and powerful strategy for immune modulation that can also be applied to other immune-driven diseases beyond cancer.

DCs have a lot of potential applications in transplantation, but there are also potential risks and issues to consider. As the most important APCs, DCs can initiate an immune response by activating T and B cells, with a risk that the immune system may still recognize the graft as foreign and initiate graft rejection [111]. Additionally, DCs are critical in the pathogenesis of graft-versus-host disease (GVHD), and animal studies have shown that the initiation of CD8^+^ T-cell-dependent acute GVHD requires host APCs, and donor APC can amplify the process, leading to tissue damage and complications [112].

The interplay between the different cells of the immune system plays a pivotal role in the survival of transplanted organs. Thus, understanding the mechanisms underlying this complex microenvironment and how immunosuppressive therapy acts on these cells is mandatory to develop a targeted therapy to decrease the rate of graft failure.

### 7.1. Posttransplant Malignancies

As previously mentioned, DC-induced immunity plays an important role in the host control of many forms of cancer. This is quite notable in the case of malignancies believed to have a strong viral etiology, given the inherently immunogenic nature of pathogen-derived (i.e., nonself) antigens that may be latently expressed in cancer cells to maintain the transformed state. 

Research indicates a general increase in cancer risk of two- to four-times following organ transplantation. The mechanisms driving this oncogenesis include prolonged immunosuppression, which decreases immune surveillance of abnormal cells, as well as opportunistic infections posttransplantation, notably viral infections. In patients receiving high-dose immunosuppressive regimens for graft maintenance, one would predict that in the absence of tumor-specific T-effector cell priming/activation, these forms of cancers would occur as posttransplant malignancies in patients who are seropositive for reactivity against “oncoviruses”, or those seronegative patients who received organ grafts from seropositive donors. Indeed, one of the major forms of transplant-related malignancy is skin cancer and posttransplant lymphoproliferative disorder (PTLD) following solid organ transplantation [113,114,115,116]. Squamous cell carcinoma (SCC) and basal cell carcinoma (BCC) represent almost 90% of all non-melanoma skin cancers [117,118]. Compared with liver transplant recipients, heart, lung, and renal transplanted patients have a higher incidence of skin cancers, which could be related to the reduced cumulative dosage of immunosuppressive drugs in liver transplantation. DCs, along with macrophages, are frequently described as well-represented cell populations in the BCC microenvironment, although they are less prevalent than T cells and may have a protective role. Higher levels of S-100, a marker of LCs, were associated with less aggressive BCC. In contrast, DCs lacking maturation markers, including CD40, CD83, and LAMP, have been reported in the BCC microenvironment, resulting in a pro-tumorigenic function and the induction of T-cell tolerance [119].

Minohara et al. suggested that targeting mregDCs, which are characterized by the expression of IL23A and IL12B, could offer a novel strategy for immunomodulation in SCC. This is attributed to their crucial involvement in inducing IL17 production by T cells. Therefore, to control the Th17/Treg ratio in the TME [120], patients with PTLD exhibit peripheral populations of malignant CD20^+^ B cells that have been transformed by Epstein-Barr virus (EBV), with a major predisposition factor being a mismatch in the EBV serostatus between the graft donor and the recipient. The incidence of EBV-related PTLD is estimated at 1–5%, with the highest incidence (5–20%) in recipients with intestinal and multivisceral transplants and a lower incidence (1–5%) in kidney transplanted patients [121]. Lim et al. demonstrated that kidney and heart transplant recipients have lower numbers of circulating pDC precursors. Interestingly, the defect in the antiviral response in kidney transplanted patients was due to a reduced number of cells rather than their functional dysfunction. In an animal model, EBV-stimulated pDCs produced IFN-α and induced the activation of NK cells and CD3^+^ T cells via toll-like receptor 9; instead, the depletion of pDCs led to enhanced mortality for EBV infection [122]. However, studies have shown that the production of IL-10 is increased in EBV-stimulated pDCs, thereby hindering the immunorecognition of virus-infected B cells. Moreover, IL-10 is not only linked to a decreased expression of costimulatory markers, activation, and proliferation of cells but also to a decreased production of INF-α and INF-γ by pDCs, impairing antiviral responses [123]. Additionally, Struthers et al. found significantly greater numbers of pDCs in samples of early lesions of B-PTLDs compared with polymorphic and monomorphic tumors, underlying the pathogenic role of pDCs in PTLD [124]. The effectiveness of current treatments for PTLD in transplanted patients is limited and can be associated with toxicities or rejection. mTORi has been demonstrated to suppress EBV-related tumor growth in vitro and in vivo but also showed high variability in efficacy and clinical benefits. Sang et al. showed that the inhibition of either phosphoinositide 3-kinase (PI3K) or AKT serine-threonine protein kinase reduced the proliferation of EBV^+^ B-cell lines from PTLD patients synergistically with the use of RAPA [125]. 

The development of CTLs capable of recognizing EBV, CMV, and adenovirus has been reported using a recombinant adenovirus to infect B cells infected by EBV and stimulate the growth of CTLs, which showed the ability to reduce disease symptoms in PTLD patients. Regarding DCs, an adenoviral vector encoding latent membrane protein 2 (LMP2) was used to develop a large number of CD4^+^ and CD8^+^ cells to restore the pool of lymphocytes in patients who have been heavily treated with cytotoxic drugs [126].

In addition, other DNA viruses have been reported to induce the development of malignancies with increased frequencies in the posttransplant setting in solid organ recipients, including cervical carcinoma and cervical intraepithelial neoplasia HPV-related (human papillomavirus), Kaposi’s sarcoma (human herpes virus-B-related), and hepatocellular carcinoma (hepatitis B-related and hepatitis C-related), among others. Compared with age-matched individuals who have not undergone transplantation, solid organ transplant recipients have a higher risk of developing HPV-associated malignancies [127]. Several immunosuppressive strategies have been adopted by high-risk HPV to escape immunosurveillance. One of them is the ability of some HPV oncoproteins, such as E6 and E7, to alter the recruitment and localization of epidermal DCs. E6 and E7 oncoproteins trigger cell transformation in infected basal keratinocytes by interfering with the host cell cycle regulatory proteins, and their expression was associated with impaired differentiation of monocytes into LCs and the depletion of intraepithelial LCs. Moreover, DCs have a limited ability to stimulate CTL responses because of the HPV-related impaired DC maturation, leading to lesion progression [128].

Among the viruses that can persist lifelong in a latent form, human herpes virus-8 (HHV-8) has a critical role in immunosuppressed patients because of the possibility of lytic reactivation and uncontrolled proliferation of latently infected cells. Kaposi’s sarcoma (KS) is a multicentric neoplasm that develops when lymphatic-endothelium-derived cells are infected with HHV-8. Compared with the general population, transplanted patients show a 200- to 500-fold increased risk of developing KS, and a role for immunosuppression in the development of the disease has been well established in the literature [129]. Rappocciolo et al. demonstrated that HHV-8 could infect three different types of DCS: monocyte-derived DCs, LCs, and interstitial dermal DCs. This led to a decreased ability of these cells to stimulate allogeneic CD4^+^ T-cell responses [130]. In immunocompromised patients with KS, a notable decrease in the number of LCs was observed in the epithelium of oral lesions compared with the epithelium of non-immunocompromised patients. This suggests that viral infection may not only change the function of these cells but also reduce their concentration [131].

### 7.2. Prevention and Treatment Options for Patients with Posttransplant Malignancies

When posttransplant malignancies are detected in patients, the standard treatment approach is to reduce the dose of immunosuppression to a level that may differentially maintain the allograft but may permit the recovery of a certain degree of antiviral/tumor CTL activity in these patients. Regrettably, this strategy often fails to lead to tumor regression and places the patient at a greater risk of graft rejection. What are other treatment options to consider? First, one might consider altering the conditioning regimen to either use suppressive agents that may be less prone to negating protective (at least antiviral immunity) T-cell-mediated immunity or attempting to promote active immune tolerance to the graft in other ways. In this regard, while comprehensive studies remain to be performed, there are reports that, unlike calcineurin inhibitors that have been linked to posttransplant malignancies, other immunosuppressive agents, such as MMF and rapamycin, have not. This dichotomy could be the result of direct antitumor effects linked to these latter drugs; subtle differences in how DCs and/or effector T cells are inhibited permit the functional retention of memory antiviral/tumor immunity or differential effects on Treg cells (i.e., rapamycin has been recently shown to promote the preferential expansion of Treg) [132].

While currently experimental in nature, alternative means to promote specific T-cell tolerance to the allograft via the use of anergizing (allo) DC might also be considered as a pretransplant conditioning regimen that avoids a global immunosuppressive strategy that could ablate the benefits of memory, anti-viral/tumor T cells. Given the realization that immature DCs are poor stimulators of allo-specific effector T cells and that these cells may preferentially induce Treg cells, they have been intensively investigated in tolerizing regimens in preclinical models, with extensions in the durability of allografts reported. Similarly, DCs pretreated ex vivo with IL-10, TGFβ, dihydroxyvitamin D3, corticosteroids, aspirin, and N-acetyl-Lcysteine have all exhibited deficiency in the expression of MHC class II, co-stimulatory and adhesion molecules, antigen-presenting function, and/or IL-12 production (or expression of inhibitory molecules such as ILT3 and ILT4). When applied as stimulator cells, these treated DCs promote T-cell anergy [3]. Intriguingly, a recent report suggested that specific targeting of antigen directly to murine immature DCs in vivo using a monoclonal antibody directed against DEC-205, a specific endocytic receptor expressed by immature DCs, results in antigen-specific T-cell tolerance [133]. Thus, the in vivo targeting of immature DC combined with compounds specifically inhibiting DC maturation in vivo could provide comparatively simple approaches for the induction of specific, durable tolerance in allograft recipients in a manner that may spare preexisting antitumor immunity. In cases where the patient has already developed posttransplant malignancy, or is at high risk of doing so, treatment options include adoptive immunotherapy using ex vivo generated autologous EBV-specific CTL or allogeneic CTL, cytokine therapies, antiviral agents, and, more recently, rituximab and DC cell-based therapies. In focusing on the utility of DC for treating posttransplant malignancy, several groups have demonstrated that despite the inhibitory effects of immunosuppressive agents on endogenous DC in vivo in transplant recipients, potent, mature, and immunostimulatory DCs can be generated from these individuals using various in vitro culture methods that implement proinflammatory cytokines and TLR agonists. Such DCs, when loaded with EBV antigens (as an example), have been shown to be up to 10-fold better than autologous EBV-B cell lines in their capacity to expand EBV-specific CTL ex vivo”, with the adoptive transfer of these “tumor-specific” effector T cells representing an effective passive cellular therapy for PTLD. Although logistically tenable, this approach represents a costly and patient-specific approach [134].

## 8. Conclusions

Dendritic cells play a central role in coordinating human immunity and provide a critical link between innate and adaptive immune responses. The DC/T-cell interaction can lead to two opposite outcomes: potent activation (immunogenicity) or inhibition (tolerance) of the immune response. The particular outcomes appear to be determined by the origin of DCs and their activation state, i.e., activated, mature DCs induce T-cell immunity, while resting, non-activated immature DCs can induce tolerance.

In the transplantation setting, “immunogenic” DCs may constitute potential targets for immune intervention to promote the establishment of allo-tolerance and long-term graft survival. Different mechanisms of peripheral tolerance have been attributed to particular DC subsets: T-cell anergy, induction of Treg proliferation, and immune deviation from the Th1 to Th2 response. The tolerogenic properties of DCs may be further exploited in transplantation via the infusion of immature DCs as a pretransplant conditioning regimen to prolong allograft survival in patients with posttransplant malignancies.

Given the double-edge nature of DC-induced immunity in organ graft recipients that develop posttransplant malignancy, where both tolerance (to graft) and cytopathic reactivity (against posttransplant malignancy) are desired, there is clearly a great onus on the research community to define appropriate DC-based treatments that allow for coordinated, non-pathologic co-existence of these types of T-cell (and B-cell) responses. Therefore, future studies related to the mechanisms governing the balance of in vivo DC function are needed to fully harness the clinical potential of these important cells.
